# Primary Gallbladder Small Lymphocytic Lymphoma as a Rare Postcholecystectomy Finding

**DOI:** 10.1155/2014/716071

**Published:** 2014-05-06

**Authors:** Kyriakos Psarras, Nikolaos Symeonidis, Euthymia Vlachaki, Minas Baltatzis, Georgios Papatolios, Efstathios Pavlidis, Christina Mouratidou, Ioannis Venizelos, Theodoros Pavlidis, Athanasios Sakantamis, Christina Nikolaidou

**Affiliations:** ^1^2nd Department of Propedeutical Surgery, School of Health Sciences, Aristotle University of Thessaloniki, Hippokratio General Hospital, 49 Konstantinoupoleos Street, 54642 Thessaloniki, Greece; ^2^2nd Department of Internal Medicine, School of Health Sciences, Aristotle University of Thessaloniki, Hippokratio General Hospital, 49 Konstantinoupoleos Street, 54642 Thessaloniki, Greece; ^3^Department of Pathology, Hippokratio General Hospital, 49 Konstantinoupoleos Street, 54642 Thessaloniki, Greece

## Abstract

*Introduction*. Primary lymphoma of the gallbladder is an extremely rare entity with approximately 50 cases reported so far. In many of these cases the presenting symptoms were mimicking symptomatic gallstone disease and the diagnosis was made postoperatively, especially when the preoperative imaging results were far from suspicious for malignant disease. *Patients and Methods*. We report a case of primary lymphoma of the gallbladder in an 85-year-old man with gallstone disease, who was admitted for elective cholecystectomy 2 months after an episode of acute cholecystitis and pancreatitis. Histological evaluation of the specimen revealed a small lymphocytic lymphoma of the gallbladder. This type of primary gallbladder lymphoma has not been previously reported. *Discussion*. The most common primary lymphomas of the gallbladder are MALT lymphomas and diffuse large B-cell lymphomas, although a variety of other histological types have been reported. The association of these lesions with chronic inflammation is the most convincing theory for their pathogenesis. For lesions confined to the gallbladder, cholecystectomy is considered to be sufficient, while supplementary chemotherapy significantly improves prognosis in more advanced disease.

## 1. Introduction

Extranodal non-Hodgkin lymphomas represent 40% of all malignant lymphomas, the majority of which involve B-cell clones and originate from the gastrointestinal tract (4%–20% of all non-Hodgkin lymphomas arise in this site) [1]. The stomach is more often involved, followed by the small intestine, colon, and esophagus. Primary lymphoma of the gallbladder without regional spread of the disease and without other organ involvement is extremely rare; to the best of our knowledge about 50 cases have been reported in the English literature so far [2]. Secondary infiltration of the gallbladder in patients with widespread lymphoproliferative disease is a slightly more common entity, although rare as well.

The mean age of presentation depends on the specific histological type ranging between 35 and 69 years old [2]. There are only two cases reported in 4- and 5-year-old children, the former being a lymphoblastic lymphoma of the pre-B type and the latter a T-cell non-Hodgkin lymphoma [2, 3]. A marginal female predominance has been observed [2].

Symptoms of acute cholecystitis represent the most common clinical appearance of the disease masquerading the diagnosis of lymphoma, especially if a mass in the gallbladder wall is not detected preoperatively. However, even in the case of a mass, the distinction between gallbladder adenocarcinoma and lymphoma is hardly achieved without histological confirmation.

Primary lymphomas of the extrahepatic biliary tract are even more rare presenting at younger age and most of the times are symptomatic due to bile duct obstruction [2].

## 2. Case Report

An 85-year-old man was admitted to the surgical department for elective laparoscopic cholecystectomy due to symptomatic gallstone disease. Medical history reported arterial hypertension and type II diabetes mellitus. The patient had been hospitalized in emergency 2 months before, because of severe epigastric and right upper quadrant pain radiating to the back with retching and vomitus. Laboratory findings consisted of a moderate leukocytosis (12.500/*μ*L, 78% granulocytes) and elevated serum and urine amylase. Abdominal ultrasound and MRI/MRCP revealed an edematous appearance of the pancreas, mild distention, and thickening of the gallbladder wall and an amount of bile-sludge inside the lumen which was mobile upon patient's movements. No accompanying lymphadenopathy was detected. The patient was managed conservatively and an elective late cholecystectomy was scheduled.

**Figure 1:**
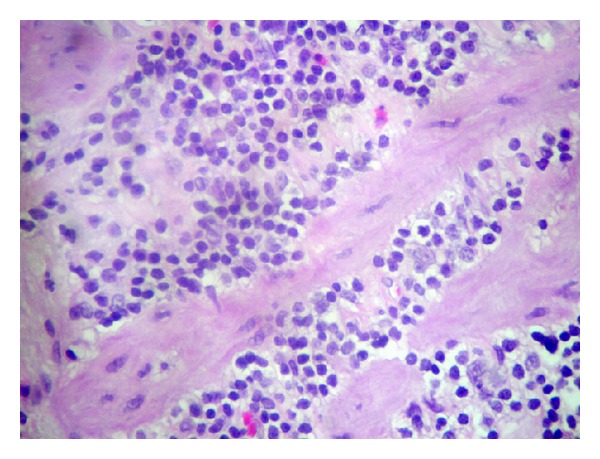
Hematoxylin-eosin stain (×400) showing small lymphocytes with round nuclei, small nucleoli, and scant cytoplasm.

**Figure 2:**
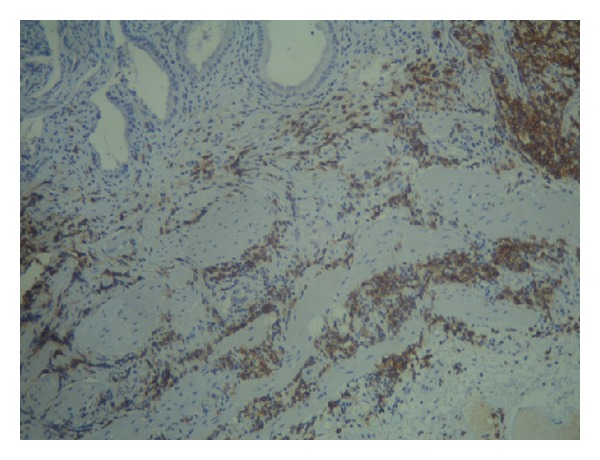
Immunohistochemistry (×200) showing CD23 positive cells infiltrating the gallbladder wall.

**Figure 3:**
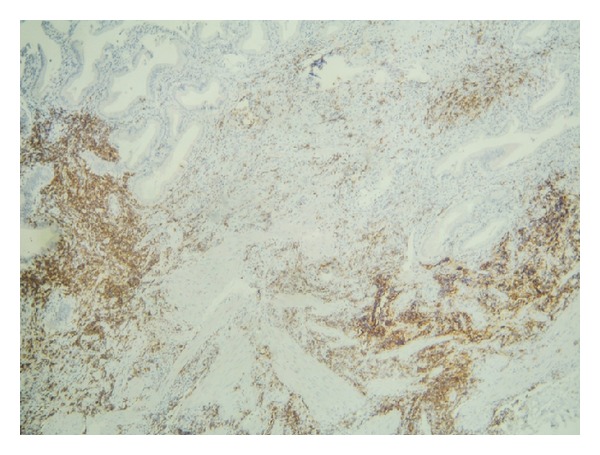
Immunohistochemistry (×40) showing CD5 positive small lymphocytic gallbladder infiltration.

**Figure 4:**
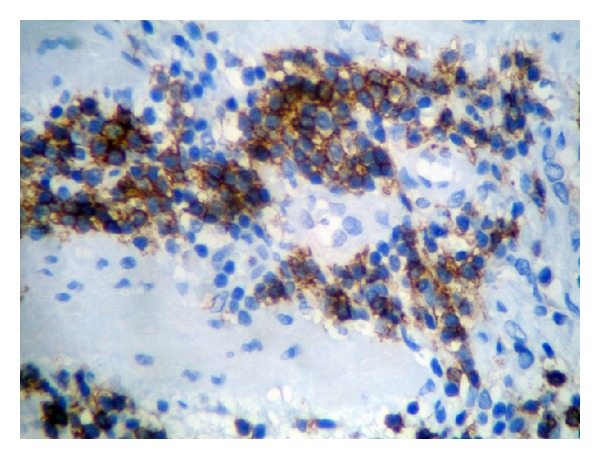
Immunohistochemistry (×400) showing CD20 positive small lymphocytes.

Two months later, the patient underwent laparoscopic cholecystectomy without intraoperative or postoperative complications. The gallbladder contained inspissated bile; the wall seemed normal on macroscopic inspection and palpation. The pathology report described a gallbladder measuring 7 × 5 cm. Microscopically, the surface epithelium was normal. In the lamina propria there was diffuse infiltration with small lymphoid cells, which had slightly irregular or round nuclei and small nucleoli. The cytoplasm was scant. There was no mitotic activity. Mild inflammatory infiltration with other lymphocytes, plasma cells, and eosinophils was also found. Immunohistochemistry revealed that the small lymphoid cells were positive for CD20, CD79a, CD5, and CD23 and negative for CD10, CD3, and Cyclin Dl. Therefore, the diagnosis of small lymphocytic lymphoma (SLL) of the gallbladder was established (Figures 1, 2, 3, and 4). The patient was readmitted in the department of hematology and additional investigations were performed. CT of the neck, thorax, and abdomen, upper and lower gastrointestinal tract endoscopies, and bone marrow biopsy were all negative, including a negative biopsy for* H. Pylori*. Bone marrow morphology, immunohistochemistry, and immunophenotyping by flow cytometry did not reveal any infiltration by B-monoclonal cells. Peripheral blood smears showed a granulocytic prevalence of 66% (WBC: 5880/*μ*L). Additionally, B-monoclonal cells in the peripheral blood were measured lower than 5 × 10^9^/L, being CD20 and CD19 positive and CD5 negative in the immunophenotyping analysis, which excluded the diagnosis of chronic lymphocytic leukemia (CLL) according to the updated WHO definition [4, 5]. It was decided that no additional treatment was necessary and the patient is under regular followup. One year after the diagnosis there are no clinical or radiological signs of recurrence.

## 3. Discussion

Primary malignant lymphoma of the gallbladder is extremely rare. Among malignant tumors of the gallbladder 98% are adenocarcinomas, while only 0.1-0.2% represents lymphomas [1]. Low-grade MALT lymphomas and high-grade diffuse large B-cell lymphomas (DLBCL) are the commonest histological types encountered in the gallbladder lymphoma [2, 6]. MALT lymphomas are usually CD5(-) and are characterized by the triad of (a) centrocyte-like cells, (b) small lymphoid cells, and (c) plasma cells, as described by Isaacson in 1987 [6, 7]. Extranodal marginal zone lymphomas (EMZL) are the most frequently reported MALT lymphomas. Both DLBCLs and EMZLs usually develop in the elderly (>70 years) and frequently coexist with gallstone disease [2]. Other rare histological types are the follicular lymphomas, the mantle cell lymphomas, the primary effusion lymphomas (PEL), the plasmablastic lymphomas (PBL) which are considered as HIV-associated lesions, the B-lymphoblastic lymphomas, the angiotropic or intravascular lymphomas, some poorly differentiated lymphocytic lymphomas, and the classical Hodgkin lymphomas [2].

SLL is defined by WHO (2008) as a low-grade neoplasm with the tissue morphology and immunophenotype of CLL, but with absence of leukemia [8]. It accounts for about 4-5% of non-Hodgkin's lymphomas and the median age of presentation is 60 years. Histologically they appear as small round lymphocytes interspersing with larger cells with prominent nuclei [9]. SLL is an indolent disease with a very good prognosis and therefore it is frequently indistinguishable from the benign monoclonal B-cell lymphocytosis, which needs no treatment. Research for specific histological criteria in order to make a distinction between these 2 entities is currently in progress [8]. Only 3 cases with CLL involving the gallbladder have been reported [10–12]. Recently, Imenpour et al. reported a case of SLL/CLL of the gallbladder accompanied with abdominal, thoracic, and axillary lymph node involvement [13]. No other case of primary gallbladder SLL has been reported so far after a thorough investigation of the literature.

The pathogenesis of SLL in the gallbladder is obscure. Taking into account our patient's history of cholecystitis we can speculate that it may be comparable with the pathogenesis of MALT lymphomas, which arise in lymphoid tissue in response to either infectious conditions or autoimmune diseases [14, 15]. Specifically in the gallbladder, chronic inflammation possibly provides a good substrate for primary lymphomas [16]. Tomori et al. reported that lymphoid follicles are normally present in the gallbladder wall and also that lymphoid proliferation and MALT lymphomas are associated with positive bile cultures and gallstones [17]. Bisig et al. postulated that mechanical irritation rather than infectious conditions is responsible for MALT gallbladder lymphomas, based on the fact that infected bile was rarely detected in reported cases [18]. Nevertheless, regardless of the specific mechanism that causes inflammation, cholecystitis induces lymphocyte migration to the gallbladder mucosa, resulting in the formation of secondary follicles. Continuous antigenic stimuli may cause chromosomal translocations leading to a fusion protein, which inhibits apoptosis and results in antigen-independent proliferation [19]. Bisig et al. detected a specific translocation* t(11;18)(q21;q21)* in an EZML type of primary gallbladder lymphoma, leading to the fusion of the apoptosis inhibitor-2 (API2) gene with the MALT lymphoma-associated translocation (MALT1) gene [18].

Clinically, gallbladder lymphomas may mimic acute or chronic cholecystitis with or without cholelithiasis. Mani et al. reported a series of 14 patients with primary gallbladder lymphoma, 10 of which presented with symptoms of cholecystitis [2]. Two patients from the same series had a palpable gallbladder mass, while cholelithiasis was detected in 4 cases. The majority of other isolated cases do not demonstrate an exception to this rule; therefore, no characteristic clinical features have been described. SLL/CLLs of the gallbladder may also present with acute cholecystitis as in our case [12].

Radiological features of gallbladder lymphomas are highly associated with the histological type. On CT, high-grade lymphomas, such as DLBCL, demonstrate a tendency to form a solid mass or an irregular thickening in the gallbladder wall, whereas low grade lymphomas (MALT, follicular, or SLL) may present as a slight thickening of the wall. Furthermore, in early stages of the disease, the lesion is confined to the submucosal layer leaving the mucosa intact. This finding is of great importance regarding the differential diagnosis from gallbladder adenocarcinoma [1, 20, 21]. On MRI, gallbladder lymphoma lesions show low signal intensity on fat-suppressed T_1_-weighted sequences and high signal intensity on fat-suppressed T_2_-weighted sequences compared with those of liver parenchyma. On the latter sequences the signal of the wall is homogenous and slightly hypointense compared with gallbladder carcinoma [22]. It is worth mentioning that in our case the lesion was not detected in any imaging studies.

Trying to estimate the necessity or routine gallbladder histopathology, Huang et al. reported a series of 1452 cholecystectomies that included only 6 carcinomas and 1 lymphoma discovered upon pathological evaluation [23]. Based on the overall low incidence of the gallbladder malignancy and also on the fact that in all malignant cases of his series the lesions were suspected pre- or intraoperatively, he proposed that a selective rather than a routine histological examination would result in reduced demands in the histopathology department without compromising patients' safety. However, the presence of some incidental cases, without any clinical or radiological suspicion as in our case, proves that several cases could be misdiagnosed if routine examination was not accomplished. Cost-effective practice frequently gives rise to ethical concerns and induces a great amount of controversy, especially when human life is the matter.

In case of incidental diagnosis of primary gallbladder lymphoma all patients should undergo a complete staging workup in order to determine the extent of the disease, as well as a follow-up surveillance. If the lesion is localized in the gallbladder (as in our case) cholecystectomy is considered to be sufficient. Otherwise, patients should undergo neoadjuvant therapy and in case of SLL/CLL the combination of oral fludarabine and rituximab has been recently proposed as an effective initial treatment, which prolongs progression free interval and overall survival [24]. Chlorambucil, cyclophosphamide, and steroids have also been used with satisfactory response rates. Radiation therapy has also been proved valuable. The prognosis of SLL is very good (mean survival 75 months after diagnosis [25]) and the majority of patients' deaths are attributed to other causes.
